# Reductions in malaria and anaemia case and death burden at hospitals following scale-up of malaria control in Zanzibar, 1999-2008

**DOI:** 10.1186/1475-2875-10-46

**Published:** 2011-02-18

**Authors:** Maru W Aregawi, Abdullah S Ali, Abdul-wahiyd Al-mafazy, Fabrizio Molteni, Samson Katikiti, Marian Warsame, Ritha JA Njau, Ryuichi Komatsu, Eline Korenromp, Mehran Hosseini, Daniel Low-Beer, Anders Bjorkman, Umberto D'Alessandro, Marc Coosemans, Mac Otten

**Affiliations:** 1 World Health Organization, Global Malaria Programme, 20 Avenue Appia 1211 Geneva 27, Switzerland; 2 World Health Organization, Inter-Country Support Team for Southern and Eastern Africa, Regional Office for Africa (AFRO), Harare, Zimbabwe; 3 Zanzibar Malaria Control Programme, Ministry of Health, Zanzibar; 4 Global Fund for AIDS, TB and Malaria, Geneva, Switzerland; 5 Dept. of Public Health, Erasmus MC, University Medical Center Rotterdam, The Netherlands; 6 World Health Organization, Country Office, Dar-es-Salaam, Tanzania; 7 Department Parasitology, Institute of Tropical Medicine, Nationalestraat 155, B-2000, Antwerp, Belgium; 8 Department of Medicine, Karolinska University Hospital, Solna, Sweden

## Abstract

**Background:**

In Zanzibar, the Ministry of Health and partners accelerated malaria control from September 2003 onwards. The impact of the scale-up of insecticide-treated nets (ITN), indoor-residual spraying (IRS) and artemisinin-combination therapy (ACT) combined on malaria burden was assessed at six out of seven in-patient health facilities.

**Methods:**

Numbers of outpatient and inpatient cases and deaths were compared between 2008 and the pre-intervention period 1999-2003. Reductions were estimated by segmented log-linear regression, adjusting the effect size for time trends during the pre-intervention period.

**Results:**

In 2008, for all age groups combined, malaria deaths had fallen by an estimated 90% (95% confidence interval 55-98%)(p < 0.025), malaria in-patient cases by 78% (48-90%), and parasitologically-confirmed malaria out-patient cases by 99.5% (92-99.9%). Anaemia in-patient cases decreased by 87% (57-96%); anaemia deaths and out-patient cases declined without reaching statistical significance due to small numbers. Reductions were similar for children under-five and older ages. Among under-fives, the proportion of all-cause deaths due to malaria fell from 46% in 1999-2003 to 12% in 2008 (p < 0.01) and that for anaemia from 26% to 4% (p < 0.01). Cases and deaths due to other causes fluctuated or increased over 1999-2008, without consistent difference in the trend before and after 2003.

**Conclusions:**

Scaling-up effective malaria interventions reduced malaria-related burden at health facilities by over 75% within 5 years. In high-malaria settings, intensified malaria control can substantially contribute to reaching the Millennium Development Goal 4 target of reducing under-five mortality by two-thirds between 1990 and 2015.

## Background

Before scale-up of malaria control activities in 2004-2005, malaria was the leading cause of illness and death in Zanzibar, accounting for more than 50% of in-patient cases and deaths in hospitals and nearly 40% of all out-patient consultations. Endemicity was classified as moderate to high, with transmission occurring for more than 6 months per year [1,2]. In 2003, malaria prevalence in sampled 60 clusters of Shehia (the smallest administrative unit with estimated population of 3,000) ranged between 9% and 50% in children under five years of age and other age groups, with *Plasmodium falciparum *the predominant malaria species [2]. In the year 2000, chloroquine, the first-line drug then, had a treatment failure of 60% at 14 days follow up [3].

Between end-2003 to 2006, in response to the enormous malaria burden and to the inadequacy of chloroquine treatment, the Zanzibar Ministry of Health and Social Welfare and partners initiated a concerted effort to accelerate malaria control activities. With support of the Global Fund to Fight AIDS, Tuberculosis and Malaria (starting in 2005), the USA President's Malaria Initiative, the Italian Cooperation through WHO, and other partners, the government scaled up four key malaria control interventions: deployment of artemisinin-based combination therapy (ACT), distribution of insecticide-treated nets (ITNs), including long-lasting insecticidal nets (LLINs), indoor residual spraying (IRS) and intermittent preventive treatment with sulphadoxine-pyrimethamine for pregnant women (SP-IPTp).

In September 2003, artemether-lumefantrine was introduced in all public health facilities, free-of-charge, as the first-line treatment for uncomplicated malaria, regardless of age (Zanzibar Ministry of Health and Social Welfare, unpublished report, 2002), while SP-IPTp was initiated in 2004. From 2004, ITNs were distributed for free, to pregnant women and children under five years of age attending antenatal clinics in public health facilities; LLINs followed by mid-2005. IRS with lambdacyhalothrin was implemented since June-July 2006, covering nearly all households (one round in 2006, two rounds in 2007 and one round in 2008), with the exception of the main town's centre. In May 2007, 74% children under-5 and 73% pregnant women used an ITN. In addition, nearly 95% of houses had been sprayed with an insecticide during the previous six months [4].

This study reports on the impact of the interventions' scale up in six out of seven Zanzibar's in-patient health facilities, based on analysis of time trends in malaria-related and overall child and adult morbidity and mortality over a 10-year period (1999-2008). Results are discussed in light of international goals and targets for reducing malaria and improving child health at a population level.

## Methods

### Sample of hospitals

Out of Zanzibar's 126 total health facilities, seven are in-patient facilities; this analysis covers six of them: Chakechake, Mkoani and Wete district hospitals; and the three large primary health care centres with in-patient services (Kivunge and Makunduchi on Unguja island, and Micheweni centre on Pemba island). Zanzibar's main referral hospital was excluded because surveillance data for the past years were not available.

### Indicators

Monthly records of outpatient cases, inpatient cases and inpatient deaths, stratified by age <5 years old and ≥5 years old, were coded according to recorded diagnosis, i.e. malaria, anaemia and 'other conditions'.

Inpatient malaria cases and deaths include those that were parasitologically-confirmed and those admitted based on clinical judgement. For malaria outpatient cases, analyses were done both for cases with a confirmed parasitological diagnosis and for the total number of cases, including those diagnosed presumptively. To facilitate interpretation of trends in outpatient cases, we computed slide positivity rate, by dividing the number of slides positive for malaria by the total number of slides examined.

Cases and deaths attributed to anaemia, to which malaria is an important contributor in high-endemic settings [5], were analyzed as a proxy indicator of malaria-related burden.

Possible confounding of the interventions' effect by external, non-program related determinants was explored by analysing the time trend in monthly rainfall and temperature, the principal short-term predictor of malaria transmission intensity and burden [6].

### Statistical analysis of time trends

Impact was evaluated by comparing 2008 indicators with those in the pre-intervention period 1999--2003 using a segmented regressive model of an interrupted time series[7]. Impact is defined as the ratio between the observed and a counterfactual estimated indicator levels in 2008, the latter calculated assuming a (hypothetical) continuation of the pre-intervention time trend throughout 2008, thereby adjusting for time trends occurring independent of the intervention's effect. The 95% confidence intervals (CI) around impact estimates were computed based on CIs around regression coefficient estimates.

Changes over time in proportions of cases or deaths due to malaria or other causes were evaluated by Chi-squared test. The exact Fisher test was used where the chi-squared test was not suitable due to small numbers, i.e. where the expected value in any of the cells of a contingency table was below 5.

## Results

**Figure 1:**
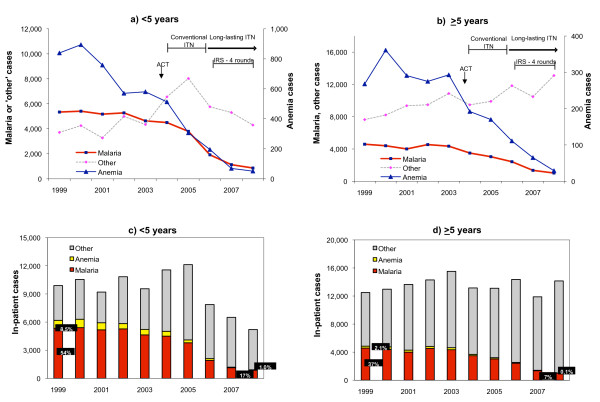
**In-patient cases due to malaria, anaemia and other causes in children under 5 years and >5 years old, 6 hospitals in Zanzibar**.

Malaria in-patient cases were fairly stable between 1999 and 2002 but declined gradually thereafter, both among children under-5 (Figure 1a) and in the older age group (Figure 1b). A similar decline was seen for anaemia cases, although among children under-five this decline started already around the year 2000. In contrast, hospitalizations due to other causes tended to increase throughout the evaluation period. This resulted in a stable number of total hospitalizations in the older age group (Figure 1d). Among children under-five, the number of hospitalizations decreased, while the percentage of malaria cases decreased from 54% in 1999 to 17% in 2008 (Figure 1c).

**Figure 2:**
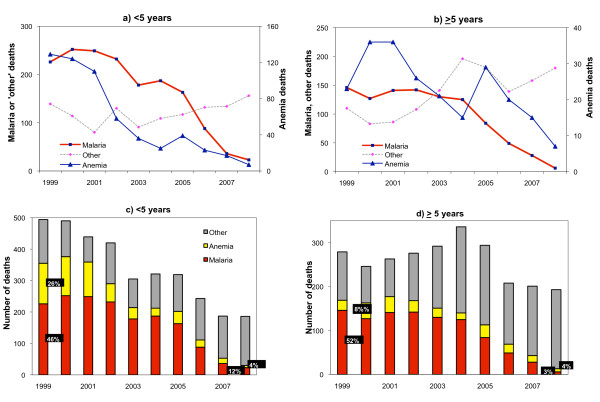
**In-patient deaths due to malaria, anaemia and other causes in children under 5 years and >5 years old, 6 hospitals in Zanzibar**.

Hospital deaths showed similar time patterns across causes for both age groups (Figure 2a and 2b). Among under-fives, among all-cause deaths, the proportion due to malaria fell from 46% in 1999 to 12% in 2008 (p < 0.01)(Figure 2c) and that due to anaemia from 26% to 4% (p < 0.01). Similarly, in the older age group the proportion of malaria deaths decreased from 52% in 1999 to 4% in 2008 (p < 0.01)(Figure 2d) and that due to anaemia from 8% to 4% (p < 0.05). Deaths due to other causes did not change or rather increased, with no consistent difference in the time trend before and after 2003.

**Figure 3:**
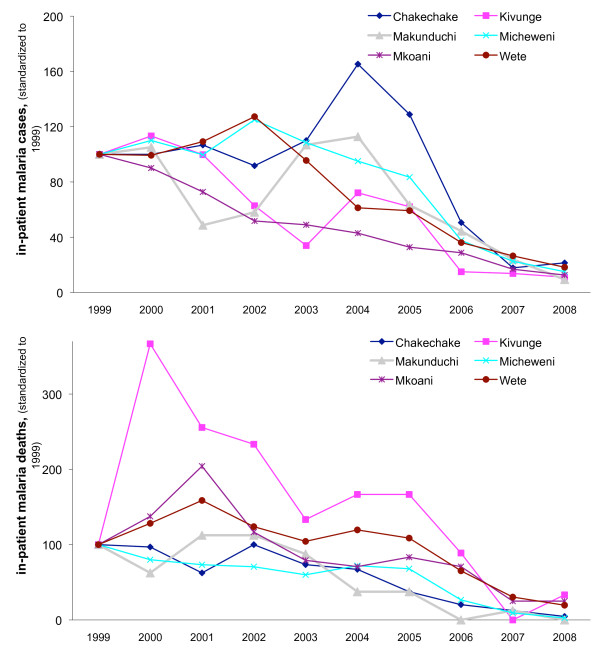
**In-patient (a) cases and (b) deaths due to malaria, children under-5 years, in individual hospitals**.

Within each of the six individual health facilities, similar declines were observed in the number of malaria in-patient cases (Figure 3a), although the onset of malaria-related reductions varied among facilities. In Chakechake hospital, malaria in-patient cases peaked in 2004 and then decreased 3-fold by 2007--2008 as compared to the 1999 level. Kivunge and Makunduchi showed similar but lower peaks in 2004 with subsequent declines to below the 1999 level. In the other 3 health facilities, no major peaks were observed: malaria cases were stable initially, declining from 2003 onwards in Wete and Micheweni, and declining throughout the 10-year period in Mkoani. Malaria deaths also declined in each of the individual hospital, starting in 2002--2003 and reaching numbers well below the 1999 levels by 2008 (Figure 3b).

**Figure 4:**
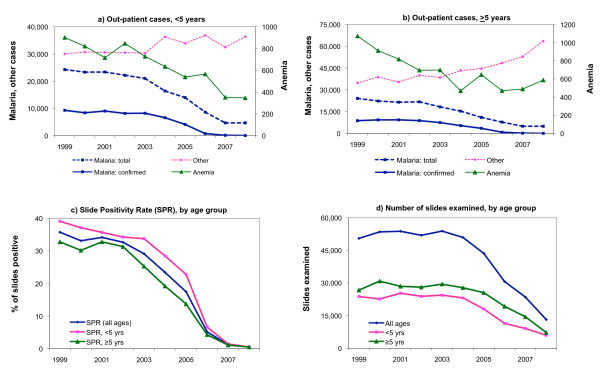
**Out-patient cases due to malaria, anaemia and other causes, (a) children under-5 and (b) older ages; (c) malaria slide positivity rates and (d) numbers of slides examined for malaria, 6 hospitals in Zanzibar**.

Malaria out-patient cases totalled 24,000 in 1999, among children under-5, out of which 9,000 were microscopically confirmed (Figure 4a). These numbers declined from 2003 onwards, reaching 5,000 total cases of which only 31 were confirmed in 2008. The decline was more marked for confirmed cases and this was paralleled by a similar decrease in the slide positivity rate (Figure 4c). Numbers of slides examined also fell, in line with the number of suspected cases; both of these indicators decreasing around three-fold between 1999 and 2008 (Figure 4d). A similar pattern was observed for the older age group (Figure 4b). The number of inpatient anaemia cases declined in a similar manner, with about a 2-fold decrease by 2008 compared to 1999-2001 levels.

Adjusting for pre-intervention time trends, malaria-attributed in-patient and out-patient cases and deaths decreased by 76% or more by 2008 compared to 1999-2003, both age groups (Table 1; p < 0.025 for each indicator). In-patient anaemia cases decreased by 85% and 90% among under-fives and over-fives, respectively (p < 0.025), with non-significant decrease for anaemia deaths (by 38% in under-fives and 66% for older ages). In contrast, no difference (and sometimes an increase) was observed in the number of cases and deaths due to other causes, without any consistent difference in time trends before and after 2003 (Table 1).

**Table 1:** Malaria and non-malaria cases and deaths in 2008 compared to the pre-intervention period 1999--2003, in six out of seven hospitals in Zanzibar

		MALARIA			ANAEMIA		
	**Age (years)**	**Average 1999-2003**	**2008**	**% decline (95 CI)**	**Average 1999-2003**	**2008**	**% decline (95 CI)**

**In-patient cases**	**<5**	5,166	861	80 (44 - 93)‡	728	51	85 (39 - 97)‡
	**5+**	4,376	1,017	76 (33 - 91)‡	298	29	90 (38 - 98)‡
	**All**	9,542	1,878	78 (48 - 90)‡	1,026	80	87 (57 - 96)‡
**Out-patient cases (confirmed)**	**<5**	8,637	31	99.6 (93 - 99.98)‡	804	347	45 (-60 - 81)
	**5+**	8,738	36	99 (91 - 99.97)‡	840	588	-40 (-355 - 57)
	**All**	17,375	67	99.5 (92 - 99.97)‡	1,644	935	9 (-87 - 56)
**Deaths**	**<5**	227	23	86 (21 - 97)‡	91	7	38 (-1'373 - 97)
	**5+**	137	6	95 (30 - 100)‡	28	7	66 (-1'116 - 99)
	**All**	365	29	90 (55 - 98)‡	120	14	40 (-1'406 - 98)

		**OTHER**			**All-CAUSE**		

	**Age (years)**	**Average 1999-2003**	**2008**	**% decline (95 CI)**	**Average 1999-2003**	**2008**	**% decline (95 CI)**

**In-patient cases**	**<5**	4,114	4,298	21 (-243 - 82)	10,008	5,210	46 (-37 - 79)
	**5+**	9,114	13,106	13 (-60 - 53)	13,788	14,152	25 (-35 - 59)
	**All**	13,228	17,404	15 (-39 - 48)	23,796	19,362	32 (-16 - 60)
**Out-patient cases**	**<5**	30,453	36,375	-19 (-82 - 23)	54,114	41,411	17 (-38 - 50)
	**5+**	37,514	63,621	-47 (-144 - 11)	59,943	69,131	-21 (-113 - 32)
	**All**	67,968	99,996	-36 (-104 - 10)	114,057	110,542	-3 (-69 - 37)
**Deaths**	**<5**	111	156	-121 (-1'011 - 56)	430	186	14 (-116 - 66)
	**5+**	106	180	-10 (-501 - 80)	271	193	37 (-44 - 72)
	**All**	216	336	-53 (-531 - 63)	701	379	23 (-42 - 59)

Notes:

Positive values indicate a decline; negative percentages indicate an increase from 1999-2003 to 2008.

‡ Significant difference. A 95% CI not including the 0% value indicates that the difference from pre-intervention period to 2008 was statistically significant (p < 0.025).

**Figure 5:**
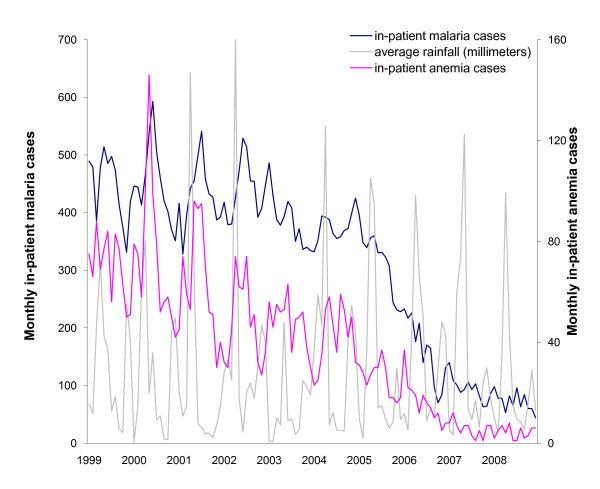
**Seasonal patterns in malaria and anaemia in-patient cases, children under-5, and monthly rainfall**.

Rainfall peaked in March--May and October--November (Figure 5). Malaria and anaemia inpatient cases varied seasonally with yearly peaks in May-June and November-December. From 2006 onwards, as malaria and anaemia cases decreased, the seasonal peaks in these hospital indicators became less pronounced.

## Discussion

This rapid impact evaluation shows that scale-up of ACT as first-line treatment of malaria, combined with vector control using ITNs/LLINs and IRS resulted a dramatic decline in the malaria burden. Within four years of intervention scale-up, malaria deaths, hospitalizations, laboratory-confirmed outpatient cases and slide positivity rates fell by 76% or more, both in children under-5 years and older age groups.

Unconfirmed (suspected) outpatient cases and numbers of slides examined also fell, but to a lesser extent (two- to three-fold), and starting later than laboratory-confirmed indicators. This suggests that the decline in confirmed malaria cases reflect a real reduction in malaria-attributable burden, and not an artefact related to changing laboratory testing patterns. The lesser decline in suspected malaria cases compared to parasitologically-confirmed cases or malaria hospitalizations and deaths confirms the notion that in endemic settings a large proportion of suspected cases is not due to malaria. This supports the WHO's 2010 recommendation for parasitological confirmation using either microscopy or RDT of all cases as a condition for ACT-based treatment, including in children under-five years in high-endemic Africa for whom presumptive treatment was recommended until 2010 - in an effort to contain costs of ACT-based treatment and to preserve ACT efficacy [8].

Hospitalizations and deaths due to anaemia fell in parallel with the malaria-attributed events, confirming the importance of malaria as an underlying cause, especially in children under-five years. These observations are consistent with previous studies that showed steady reductions in childhood anaemia in response to malaria control [5], and with observed correlation between rates of malaria and of blood transfusions in young children [9]. Anaemia represented a much larger proportion of hospital deaths than hospital admissions, e.g. among under-fives in 1999, 26% and 8.5%, respectively. This difference illustrates the poor prognosis of severe anaemia cases in Zanzibar, which is possibly due to varying availability and quality of blood transfusion services and late presentation of patients [10,11].

The decline in malaria-related burden started around 2003, when ACT was introduced, although for in-patient cases and deaths in children under five years it started slightly earlier, around 2002, possibly reflecting the increasing use, since 2001, of sulphadoxine-pyrimethamine as second-line treatment. In addition, ongoing socio-economic development and urbanization may have led to a better health over time in including malaria burden, before and during the intensified malaria control [12]. However, impact estimates using segmented log-linear regression (Table 1) were adjusted for such trends during pre-intervention period and the decrease in malaria was observed against an increase in non-malaria attendances - which may be the result of improved health services and access in recent years.

Across all 147 out-patient peripheral health facilities in Zanzibar (primary health care units), confirmed malaria cases fell by 73% between 1999 and 2008 while the slide positivity rate fell from 36% to 1.5% (Zanzibar Malaria Control Programme, unpublished, 2010). Over the same period, the slide testing rate of all outpatient consultancies in the peripheral facilities increased from 6% to 30% as a result of RDT roll-out.

In the weekly case-based surveillance system, which covers approximately one-third of health facilities, slide positivity rate was 3% in peak malaria months (April-June) during 2008 [13]. Reductions in parasitologically-confirmed malaria out-patient cases furthermore fit with results from a nation-wide population survey in May 2007, showing parasite prevalence of 0.4% in children <5 years old and 0.9% in all ages [4].

A 2007 study in North A district of Unguja using surveillance records in 13 public health facilities found a decline in under-five mortality by 52% in 2006 compared to 2003. Similarly, malaria-related admissions, blood transfusions, and malaria-attributed mortality decreased significantly by 77%, 67% and 75%, respectively, between 2002 and 2005 in children under five. While climatic conditions favourable for malaria transmission persisted throughout the observational period, additional distribution of LLINs in early 2006 resulted in a 10-fold reduction of malaria parasite prevalence [14].

In response to its current low endemicity, Zanzibar has since 2008 shifted its malaria surveillance system to weekly reporting of laboratory-based confirmed malaria cases. A Malaria Early Epidemic Detection System (MEEDS) is now operational in 52 health facilities, with the plan to expand it to all facilities by 2011. The next step for enhancing disease surveillance should be reporting and investigation of individual in-patient cases and deaths, which at low transmission levels represent a failure of the health system in adequately treating both uncomplicated and severe malaria cases. Future reporting of individual case records will also enable more precise geographical tracking of remaining transmission foci or "hotspots" and resurgences, as well as to identify risk groups and factors.

Despite Zanzibar's enormous success in reducing malaria, the risk of an explosive resurgence is still very real. This is not the first time that the islands have achieved such dramatic decline in malaria burden. In the 1970s malaria had been reduced to very low levels through IRS with dichlorodiphenyltrichloroethane (DDT), only to resurge again once partner funds decreased and IRS was stopped [15]. Aggressive malaria control activities and adequate funding therefore need to be maintained to keep the risk of malaria resurgence near to zero.

Substantial decreases in malaria burden have also been reported by other high-endemic sub-Saharan African countries that achieved high coverage of ACT, LLINs and/or IRS. In Zambia, as artemether-lumefantrine was introduced as first-line treatment, and LLINs were distributed over 2002-2008, hospital admissions and deaths, and to a lesser extent outpatient cases from malaria and anaemia, decreased substantially. Declines were more marked in children under five than among older ages, and time trends were consistent across the indicators. Repeated household surveys demonstrated parallel decreases in parasite prevalence and anaemia among children under five years. In addition, among children under five years, both all-cause mortality from household surveys and hospital-recorded deaths fell by half, in a markedly similar time pattern [16]. Importantly, these downward trends were followed by levelling off in 2009 in malaria admissions and deaths - including a major resurgence in two provinces, where parasite prevalence among children again rose according to a 2010 survey [17,18]. This rebound, possibly related to decay of insecticide and physical deterioration of ITNs distributed several years before, underscores the importance also for Zanzibar to maintain malaria control, surveillance and funding to prevent similar resurgence.

In Bioko Island, Equatorial Guinea, four years after achieving high intervention coverage, repeated household surveys found a decrease in all-cause under-five mortality of 60% from 2004 to 2008, and reductions of 70% in parasite prevalence, 90% in anaemia and 60% in reported fevers among children under-5 [19]. In Rwanda, within two years of nationwide implementation of LLIN distribution and ACT as first-line treatment, in-patient malaria cases and death in nine hospitals and 10 health centers sampled throughout 10 districts fell by 55% and 67% in children under-five, respectively. Non-malaria cases and deaths remained stable or increased [20]. Similarly, in Ethiopia, in a convenience sample of public facilities with relatively complete data, the in-patient case and death burden decreased by 73% and 62%, respectively, after the first two years of scaled-up LLIN and ACT usage [20]. In Sao Tome and Principe, after three years of intensified interventions with IRS, LLINs, ACT and SP-IPTp, malaria-attributed outpatient consultations, hospitalizations, and deaths decreased by more than 85%, 80%, and 95%, respectively, in all age groups [21]. In The Gambia, a similar retrospective analysis at four sites, showed between 2003 and 2007 a significant decline in malaria slide positivity rate, malaria admissions and malaria-related deaths. The same study also demonstrated a significant increase in mean haemoglobin concentrations for all-cause admissions (12 g/l) and age of paediatric malaria admissions (from 3.9 to 5.6 years) [9].

When interpreting the results presented, the limitations of health facility-based studies should not be underestimated [22]. Importantly, the use of a five-year period (1999-2003) as reference was not necessarily long enough to provide a stable pre-intervention baseline, given the historical resurgence of malaria in Zanzibar in the past [23]. In addition, a longer period should ideally be used as the post-intervention. Therefore, point estimates of impact based on a five-year baseline and one-year post-intervention must, therefore, be regarded as indicative, rather than precise effect sizes.

It should be also considered that time trends observed in health facility statistics may not reflect trends in malaria burden at the population level, if the completeness of case or death notifications and/or access to health facilities changes over the evaluation period, and in particular if notification fraction changed differently for malaria versus other causes of attendance. As a result, the decrease in malaria burden may be of lesser magnitude than that observed in the hospitals sampled. Of note, the reduction in all-cause under-five mortality may be lower at a population level than shown here from hospitals data, because typically in sub-Saharan African countries with stable malaria around 15-30% of deaths, not 53% as in the Zanzibar hospitals between 1999 and 2003, are due to malaria [24].

The child survival Millennium Development Goal is to reduce all-cause under-5 mortality by two-thirds by 2015 compared to 1990 baseline levels [25,26]. The new Roll Back Malaria Partnership goal is the near elimination of malaria deaths by 2015 [27]. Based on mortality estimates from 1990 to 2009, the under-5 mortality rates are now declining in all regions of the world, with declines in sub-Saharan Africa having accelerated in the 2000-2010 decade. However, a further acceleration in these declines will be needed to meet the MDG child survival goal [28].

## Conclusion

Effective malaria control measures can dramatically reduce the burden of malaria and anaemia on the health system. Ensuing reductions in all-cause under-5 mortality as a result of malaria control could play a key role in achieving MDG4 on improving child survival by 2015. Aggressive malaria control should be raised to the highest levels of public health priority in Africa and globally.

## Competing interests

The authors declare that they have no competing interests.

## Authors' contributions

MA carried out the study design, data collection, analysis and drafting and overall coordination of writing up-of the paper. AWA helped in data collection and editing; and field supervision. ASA helped in overall field coordination, reviewing the paper and programme interventions in Zanzibar. FM and AB contributed to data collection and validation, analysis and review of the paper. RN, MW and SK participated in field coordination, data collection and review of the paper. MO helped in the overall study design, data analysis and drafting of the paper. EK and MH helped in the statistical analysis and review of the paper. RK and DL provided critical review of the paper. UA and MC provided over all technical guide and reviewed the paper. All authors read and approved the final manuscript.
